# Identification of NY-ESO-1_157–165_ Specific Murine T Cell Receptors With Distinct Recognition Pattern for Tumor Immunotherapy

**DOI:** 10.3389/fimmu.2021.644520

**Published:** 2021-03-23

**Authors:** Helin Zhang, Meng Sun, Jie Wang, Bin Zeng, Xiaoqing Cao, Yi Han, Shuguang Tan, George F. Gao

**Affiliations:** ^1^Research Network of Immunity and Health (RNIH), Beijing Institutes of Life Science, Chinese Academy of Sciences, Beijing, China; ^2^University of Chinese Academy of Sciences, Beijing, China; ^3^Key Laboratory of Pathogenic Microbiology and Immunology, Institute of Microbiology, Chinese Academy of Sciences, Beijing, China; ^4^College of Life Sciences, Jiangxi Science and Technology Normal University, Nanchang, China; ^5^College of Pharmacy, Shenzhen Technology University, Shenzhen, China; ^6^Beijing Chest Hospital, Capital Medical University, Beijing, China

**Keywords:** New York esophageal squamous cell carcinoma 1, T-cell receptor, T cell receptor-engineered-T cell, tumor, human leukocyte antigen-A^*^0201

## Abstract

New York esophageal squamous cell carcinoma 1 (NY-ESO-1) is a promising target for T-cell receptor-engineered T cell (TCR-T) therapy, and targeting the human leukocyte antigen (HLA)-A2 restricted NY-ESO-1_157−165_ epitope has yielded remarkable clinical benefits in the treatment of multiple advanced malignancies. Herein, we report the identification of two NY-ESO-1_157−165_ epitope-specific murine TCRs obtained from HLA-A^*^0201 transgenic mice. NY-ESO-1_157−165_ specific TCRs were isolated after vaccinating HLA-A2 transgenic mice with epitope peptides. HZ6 and HZ8 TCRs could specifically bind to NY-ESO-1_157−165_/HLA-A2 and were capable of cytokine secretion with engineered Jurkat T cells and primary T cells upon recognition with K562 target cells expressing the single-chain trimer (SCT) of NY-ESO-1_157−165_/HLA-A2. The reactivity profiles of the HZ6 and HZ8 TCRs were found to be distinct from one another when co-cultured with K562 target cells carrying alanine-substituted NY-ESO-1_157−165_ SCTs. The binding characterization revealed that the recognition pattern of the HZ6 TCR to NY-ESO-1_157−165_/HLA-A2 was substantially different from the widely used 1G4 TCR. These findings would broaden the understanding of immunogenicity of the NY-ESO-1_157−165_, and the two identified TCRs may serve as promising candidates for the future development of TCR-T therapy for tumors.

## Introduction

Engineered T cell-based immunotherapy, mainly referred to as chimeric antigen receptor engineered T cell (CAR-T) and T-cell receptor engineered T-cell (TCR-T) therapy, has emerged as novel therapeutic strategies for a variety of tumors (1, 2). Administration of CAR-T cells has demonstrated remarkable treatment efficacy for patients with B cell malignancies, and two CAR-T drugs have been approved by the United States Food and Drug Administration (3–5). However, most tumor-specific antigens (TSA) or tumor-associated antigens (TAA) of solid tumors are located intracellularly and, are, therefore, not accessible to CAR-T cells, which usually target antigens expressed on the surface of tumor cells with a single chain variable fragment of antibodies (6). Intracellular antigens, which can be processed and presented by major histocompatibility complex (MHC) proteins, can be targeted by the TCR and, therefore, are sensitive to TCR-T cell therapy, which has been confirmed to be effective for several tumors, including melanoma and multiple myeloma (7).

New York esophageal squamous cell carcinoma 1 antigen (NY-ESO-1), a member of the cancer-testis antigen family, is considered a promising target for TCR-T cell immunotherapy. It is frequently overexpressed in various types of tumors, and its expression in normal tissue is restricted to germ cells, which can be exploited to avoid on-target toxicity as a therapeutic target (8–10). Furthermore, NY-ESO-1 has been found to be able to trigger spontaneous humoral and cellular immune responses in many tumors (11). NY-ESO-1_157−165_ (peptide sequence SLLMWITQC) has been demonstrated to be a safe and effective human leukocyte antigen (HLA)-A2 restricted antigenic epitope with therapeutic potency, which can be recognized by specific T cells (12). Several studies have identified and characterized the function of NY-ESO-1_157−165_ specific TCRs (13–15), and >30% of TCR-T cell clinical trials have targeted NY-ESO-1_157−165_ (16). Among these TCRs, 1G4, which has been extensively investigated, with clear recognition mechanisms with NY-ESO-1_157−165_ (17, 18), is currently undergoing multiple clinical trials for a range of advanced solid tumors (11), and striking clinical responses have been observed in patients with advanced multiple myeloma and synovial cell sarcoma (19–23).

Isolation of TAA-specific TCRs is the key step in the development of TCR-T therapy. Extensive screening of human T cells that recognize TAA epitopes, such as NY-ESO-1, MART-1, and neoantigens, has identified specific human TCRs for TCR-T cell therapy (24–28). However, human TCR gene-modified T cells may form unexpected TCR dimers consisting of the introduced and endogenous hetero TCR α/β pairs, which may be self-reactive and lead to lethal cytokine-driven autoimmune pathology (29). Meanwhile, it is difficult to isolate high-affinity TCRs specific for TAAs from human T-cell repertoire, which is likely due to thymic negative selection and tolerance mechanisms during T-cell development (30, 31). Murine TCRs (mTCRs) are less likely to form unexpected mixed TCRs with endogenous human TCR chains in T cells and, therefore, may enhance antitumor activity (32–34). Moreover, mTCRs lack tolerance to human TAAs; therefore, it is possible to isolate high-affinity TCRs from mice (35). mTCRs specific for MAGE, CEA, gp100, and p53 have been isolated from HLA-transgenic mice and are undergoing clinical trials (8, 36, 37). In particular, one mTCR specific for NY-ESO-1_157−165_, which was isolated from HLA-A2-transgenic mice, is now in a phase II clinical trial (NCT01967823) (34). It is necessary to isolate distinct mTCRs specific for NY-ESO-1_157−165_, which can broaden our understanding of the immunogenicity of NY-ESO-1_157−165_ and provide alternative options for NY-ESO-1_157−165_ specific TCR-T cell therapy to avoid mutational escape.

In the present study, we aimed to isolate novel and functional competent mTCRs specific to NY-ESO-1_157−165_. Two immune-dominant mTCRs were identified and TCR-T cells were generated and prepared using Jurkat T cells or primary T cells, which demonstrated that cells with these two mTCRs were capable of secreting interleukin (IL)-2 or interferon (IFN)-γ upon stimulation with specific antigens. The recognition patterns of these two TCRs were found to be distinct from one another. These findings may broaden our understanding of NY-ESO-1_157−165_ specific TCRs, and these two mTCRs may serve as promising candidates for further development.

## Results

### Single-Cell Isolation of NY-ESO-1_157–165_ Specific T Cells From HLA-A^*^0201 Transgenic Mice

**Figure 1:**
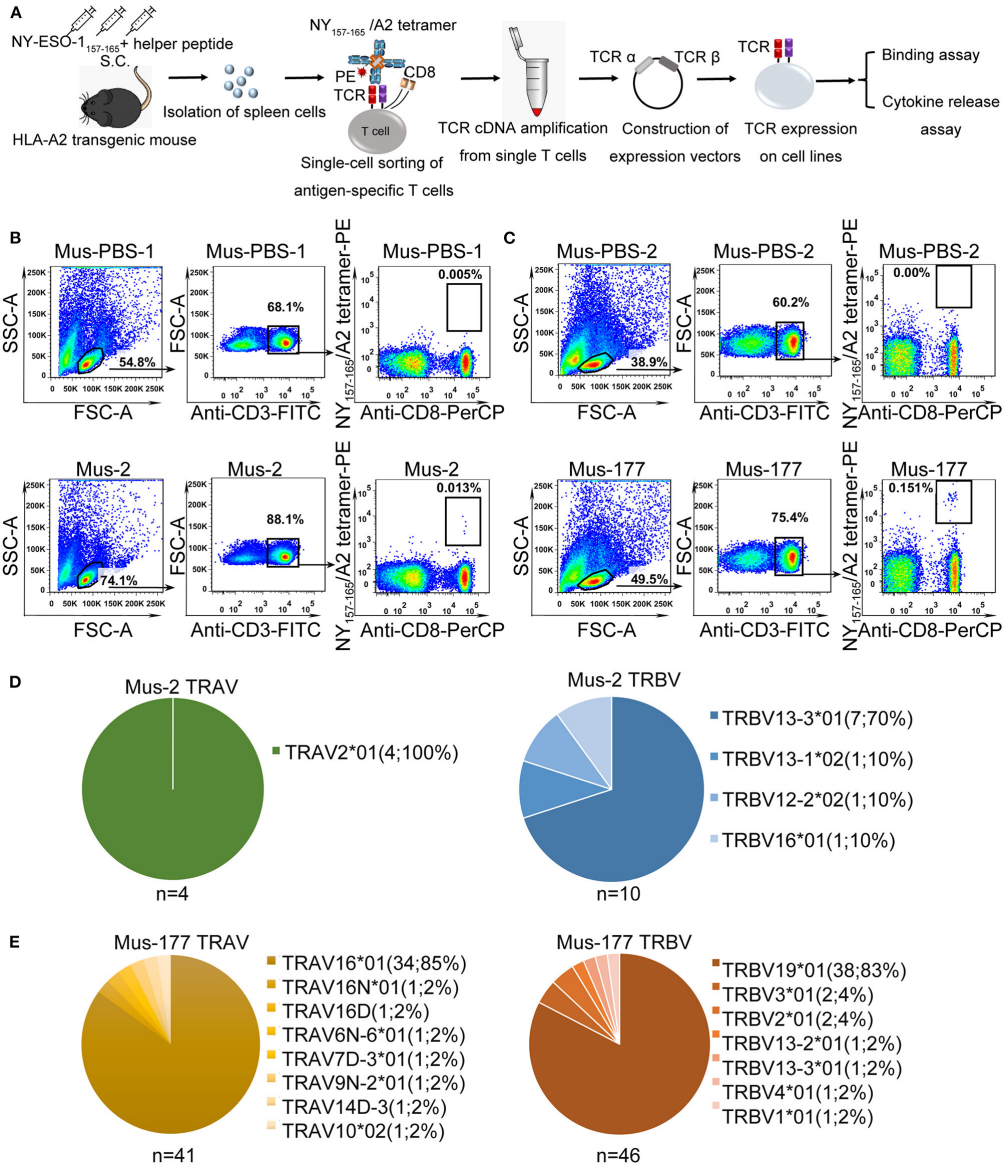
Single-cell isolation and sequencing of NY-ESO-1_157−165_-specific T cells from HLA-A*0201 transgenic mice. **(A)** Schematic of rapid cloning and functional verification strategies. **(B,C)** NY-ESO-1_157−165_/HLA-A2 tetramer-positive CD8^+^ T cells from HLA-A*0201 transgenic mice, Mus-2 **(B)** and Mus-177 **(C)** from 2 independent experiments, immunized with NY-ESO-1_157−165_, or phosphate-buffered saline (PBS) (Mus-PBS-1 and Mus-PBS-2) were analyzed with flow cytometry. Thirty-six and 48 NY-ESO-1_157−165_/HLA-A2 tetramer-positive CD8^+^ T cells from Mus-2 and Mus-177, respectively, were single-cell sorted. **(D,E)** TCR repertoire analysis of cloned NY-ESO-1_157−165_/HLA-A2 tetramer-positive CD8^+^ T cells from Mus-2 **(D)** and Mus-177 **(E)**. The number in the brackets indicates the number and frequency of T cells expressing the same TRAV or TRBV in the total number of TCRα or TCRβ clones from single cells.

The strategy used to isolate and characterize NY-ESO-1_157−165_ specific T cells is illustrated in Figure 1A. Briefly, HLA-A^*^0201 transgenic mice were immunized with a mixture of NY-ESO-1_157−165_ peptide and a helper peptide from hepatitis B virus (Supplementary Table 1). After three immunization doses, antigen-specific T cells were single-cell isolated from splenocytes with NY-ESO-1_157−165_/HLA-A2 tetramer using flow cytometry. TCR complementary DNA (cDNA) was amplified using an optimized single-cell sequencing method, cloned into an expression vector, and then transduced to HEK-293T cells or T cells for binding assay or functional studies.

NY-ESO-1_157−165_/HLA-A2 tetramer staining positive CD8^+^ T cells were identified in two mice from two independent experiments, with a total of six peptide-immunized mice: Mus-2 from the first batch and Mus-177 from the second batch (Figures 1B,C). A total of 36 and 48 CD3^+^ CD8^+^ tetramer^+^ T cells were sorted from the spleen cells of Mus-2 and Mus-177, respectively. TCR α and β variable (V) gene sequences were then amplified from single T cells using an optimized method based on the method previously described by Picelli et al. and rapid amplification of 5′ complementary DNA ends (5′ RACE) (38). The method established by Picelli et al. was used to obtain single-stranded cDNA from single T cells. Subsequently, a long-distance PCR (LD-PCR) process was performed to generate more stable double-stranded full-length cDNA. Finally, two rounds of nested PCR were used to obtain TCR α and β chain V region sequences. The efficacy of amplifying productive TCR α and β using the optimized method was 85.0% (41/48) and 95.8% (46/48), respectively, from Mus-177 (Figure 1E, Supplementary Table 2), and 40 TCRα/β cDNA pairs were obtained (Figure 1E, Supplementary Table 2). However, the amplification efficiency of the TCRs from Mus-2 was substantially lower, with only 4 Vα and 10 Vβ chain sequences (Figure 1D).

The TCR repertoire from Mus-2 and Mus-177 were then analyzed. The most frequent Vα gene segments in Mus-2 and Mus-177 were TRAV2^*^01 [4/4 (100%)] and TRAV16^*^01 [34/41 (85%)], respectively, while the most frequent Vβ gene segments in Mus-2 and Mus-177 were TRBV13-3^*^01 [7/10 (70%)] and TRBV19^*^01 [38/46 (83%)], respectively (Figures 1D,E). The TCR repertoire between Mus-2 and Mus-177 did not share any V gene segments except for TRBV13-3^*^01. The dominant TCR from Mus-2 (2-A6 in Supplementary Table 2) was renamed “HZ6 TCR,” and the most frequent TCR from Mus-177 (177-A1 in Supplementary Table 2) was renamed “HZ8 TCR.” The HZ6 or HZ8 TCR V regions were then cloned into a lentiviral expression vector with human constant regions to generate chimeric HZ6 or HZ8 TCRs, which could minimize the immunogenicity of the TCRs and limit the formation of unexpected mixed TCRs with endogenous human TCR chains.

**Figure 2:**
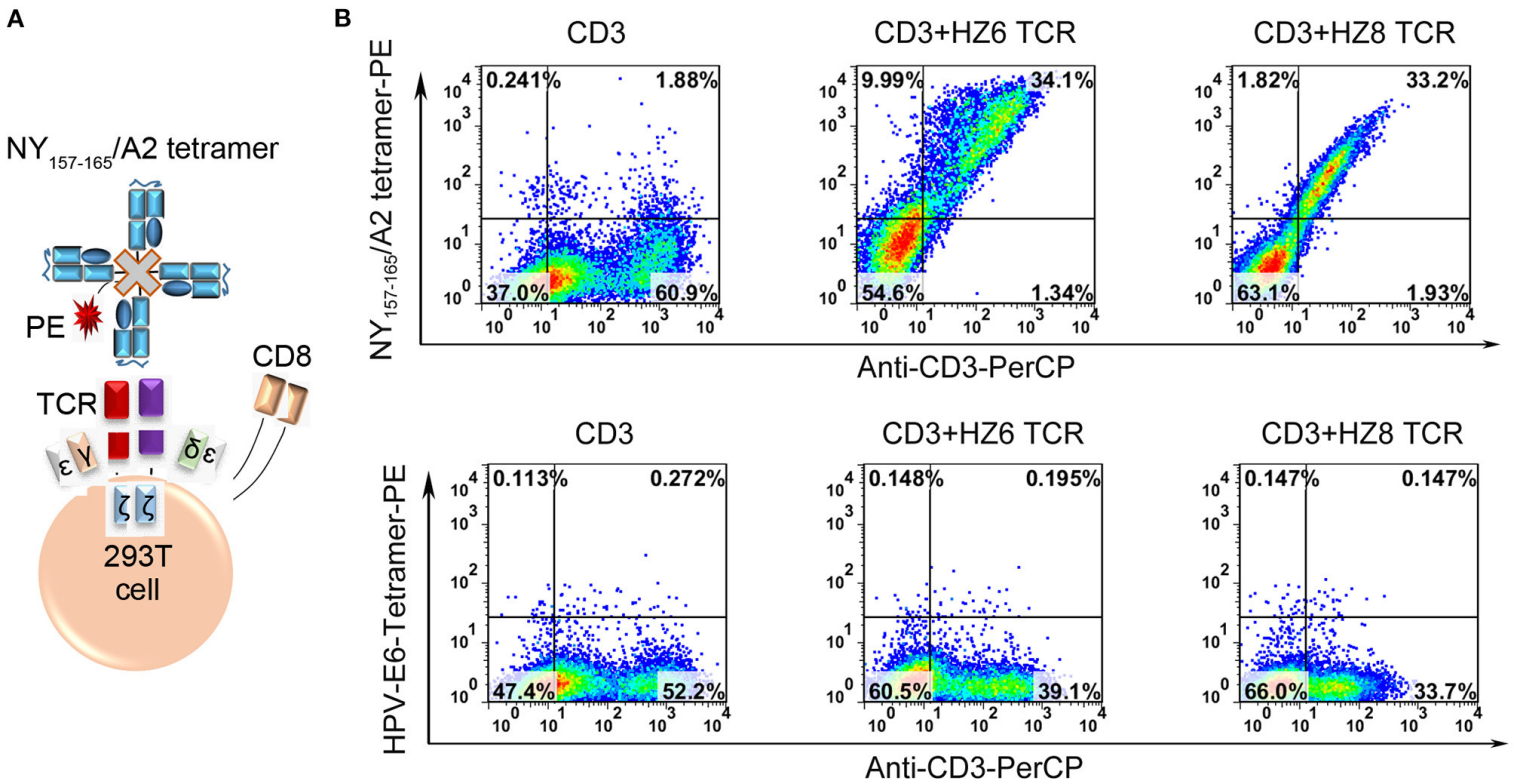
Specific binding of HZ6 and HZ8 TCRs with NY-ESO-1_157−165_/HLA-A2 in HEK-293T cells system. **(A)** Verification strategy for the detection of TCR binding specificity. Cloned TCRs and human CD3-CD8 complex were co-transfected into 293T cells. Binding of NY-ESO-1_157−165_/HLA-A2 tetramer-PE to TCRs expressed on 293T cells was analyzed with flow cytometry. **(B)** Binding of NY-ESO-1_157−165_/HLA-A2 tetramer with CD3-expressing 293T cells co-transfected with TCR and human CD3-CD8 complex constructs. Detection with HPV-E6/HLA-A2 tetramer-PE was enrolled as negative control to verify the binding specificity of HZ6 and HZ8 TCRs (lower flow chart).

To verify the antigen specificity of the HZ6 and HZ8 TCRs, flow-cytometry analysis with NY-ESO-1_157−165_/HLA-A2 tetramer was performed with 293T cells transiently co-expressing the TCR and human CD3-CD8, which were inserted into two independent expression vectors (Figure 2A) (39). Staining with anti-TCRα/β antibodies confirmed that the TCRs were expressed and exported to the cell surface (Supplementary Figure 1). The HZ6 and HZ8 TCRs exhibited NY-ESO-1_157−165_/HLA-A2 tetramer binding ability, while no non-specific binding was observed to a HLA-A2 restricted epitope from E6 protein of human papillomavirus 16 (Figure 2B). Therefore, HZ6 and HZ8 TCRs can specifically bind to the NY-ESO-1_157−165_/HLA-A2 peptide MHC (pMHC) complex.

### Distinct Response Profiles of HZ6 and HZ8 TCRs to NY-ESO-1_157–165_

**Figure 3:**
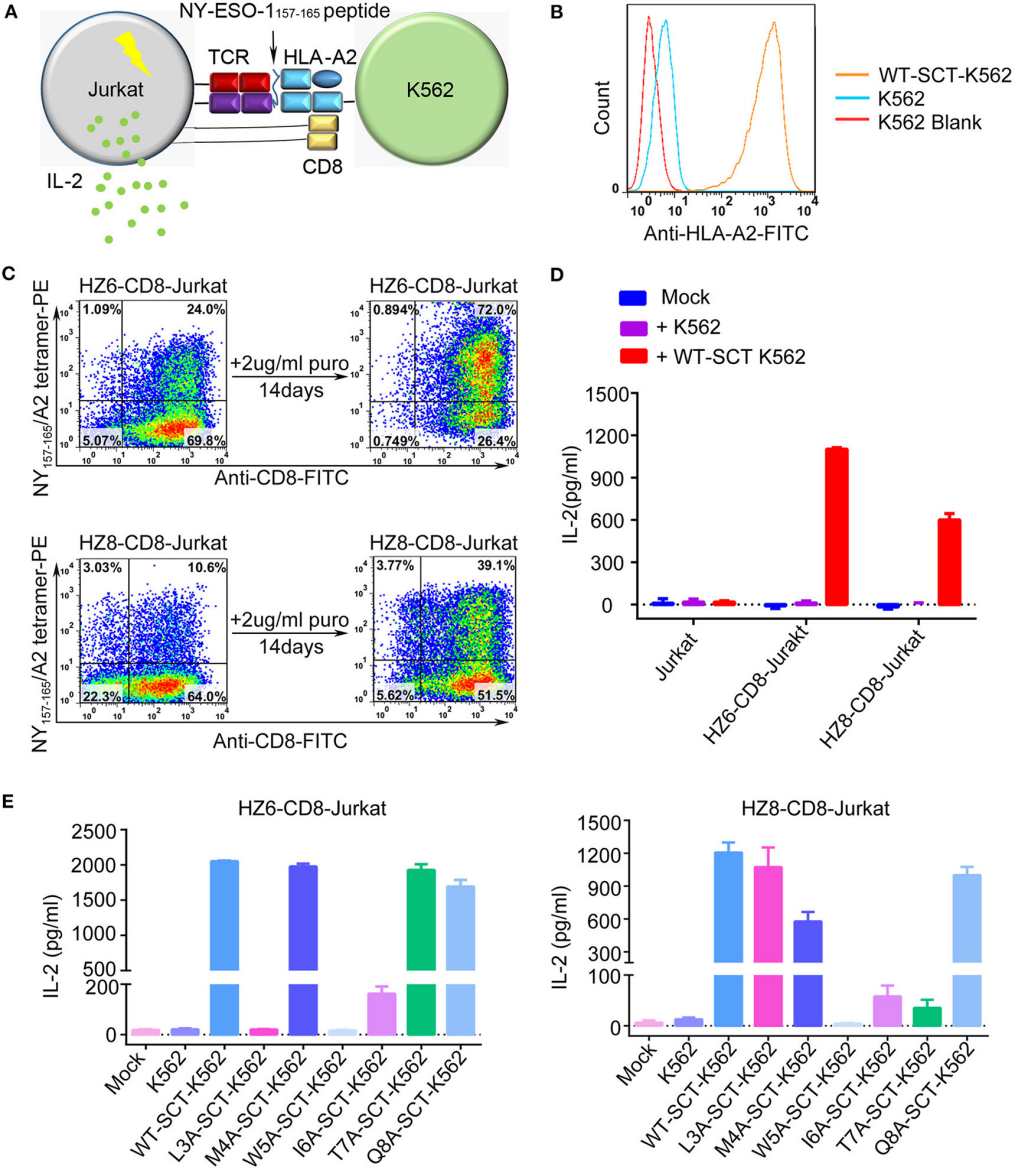
Reactivity of HZ6 and HZ8 TCR engineered Jurkat T cells to NY-ESO-1_157−165_ WT-SCT-K562 or alanine-substituted pMHC-SCTs (SCT-K562 mutants) target cells. **(A)** Schematic of the functional evaluation assay of the TCRs in Jurkat T cells. TCR engineered Jurkat T cells co-transduced with human CD3-CD8 construct were co-cultured with K562 target cells that were transduced with NY-ESO-1_157−165_/HLA-A2 single-chain trimer (SCT-K562 cells). The reactivity of TCR engineered Jurkat T cells was evaluated by detection of the IL-2 secretion after co-culturing of effector cells and target cells. **(B)** Flow cytometry histogram comparison of NY-ESO-1_157−165_/HLA-A2 expression on the surface of K562 cells transduced with NY-ESO-1_157−165_ WT-SCT (WT-SCT-K562) and K562 cells without transduction. **(C)** Flow cytometry analysis of TCR expression on Jurkat T cells transduced with TCR and CD3-CD8 before and after14 days *in vitro* culture in the presence of 2 μg/ml puromycin. **(D)** ELISA measuring secretion of IL-2 from untransduced, HZ6-CD8, and HZ8-CD8 Jurkat effector cells following 48 h co-incubation with WT-SCT-K562 target cells or K562 cells as control. **(E)** Alanine scanning approach for HZ6 and HZ8 TCR in Jurkat T cells. HZ6-CD8-Jurkat cells or HZ8-CD8-Jurkat cells were co-cultured with WT-SCT-K562 or alanine-substituted pMHC-SCTs K562 (SCT-K562 mutants) cells. IL-2 concentrations in the supernatant were measured by ELISA. For **(D,E)**, the data is a representative of 3 independent experiments with two technical replicates. Means ± SD for a representative experiment are shown.

Induction of cytokine production by the HZ6 and HZ8 TCRs was further investigated in Jurkat T cells co-transduced with TCR and CD3-CD8 constructs (TCR-CD8-Jurkat) as effector cells, while K562 cells transfected with pMHC single-chain trimer (SCT) of NY-ESO-1_157−165_/HLA-A2 construct (SCT-K562) were used as target cells (Figures 3A–C). The TCR-CD8-Jurkat effector cells and SCT-K562 target cells were co-incubated, and the secreted IL-2 in the supernatants was tested using ELISA. The results revealed that specific release of IL-2 could be observed with HZ6 and HZ8 TCR-CD8-Jurkat cells co-cultured with SCT-K562 target cells, whereas no substantial IL-2 secretion was observed in non-transduced Jurkat T cells or TCR-CD8-Jurkat cells co-cultured with K562 cells (Figure 3D). Therefore, both HZ6 and HZ8 TCRs were competent in inducing IL-2 secretion in Jurkat T cells upon recognition with NY-ESO-1_157−165_/HLA-A2.

An alanine scanning approach was used to investigate functional profiles of HZ6 or HZ8 TCR engineered effector T cells upon recognition with NY-ESO-1_157−165_ peptide, with amino acids from position 3 to 8 of the NY-ESO-1_157−165_ peptide (potentially exposed residues presented by HLA-A2) being sequentially replaced with alanine (i.e., “A”) in the pMHC-SCT construct (Supplementary Table 1). K562 cells stably expressing alanine-substituted pMHC-SCTs (SCT-K562 mutants) were similarly obtained by lentivirus transduction with wild-type (WT) SCT-K562 cells (Supplementary Figure 2). Investigation of the reactivity of HZ6 and HZ8 TCR-CD8-Jurkat T cells against the SCT-K562 mutants revealed that the recognition patterns of these two TCRs were distinct from one another (Figure 3E, Supplementary Table 3). The results revealed that W5A and I6A mutations in pMHC-SCTs substantially attenuated IL-2 secretion capacity for both HZ6 and HZ8 TCR-CD8-Jurkat cells, while M4A and Q8A mutations did not affect IL-2 secretion for either TCR. However, L3A mutated SCT-K562 completely attenuated the IL-2 secretion capacity of the HZ6 TCR-CD8 Jurkat cells, while IL-2 secretion levels in HZ8 TCR-CD8 Jurkat remained comparable to that in WT-SCT-K562 cells. In contrast, the T7A mutation exerted substantial influence on HZ8 but not on HZ6 TCR. Therefore, the determinant residues in NY-ESO-1_157−165_ for the reactivity of HZ6 TCR varied to that of HZ8.

### Functional Evaluation of HZ6 TCR-Engineered Primary T Cells

**Figure 4:**
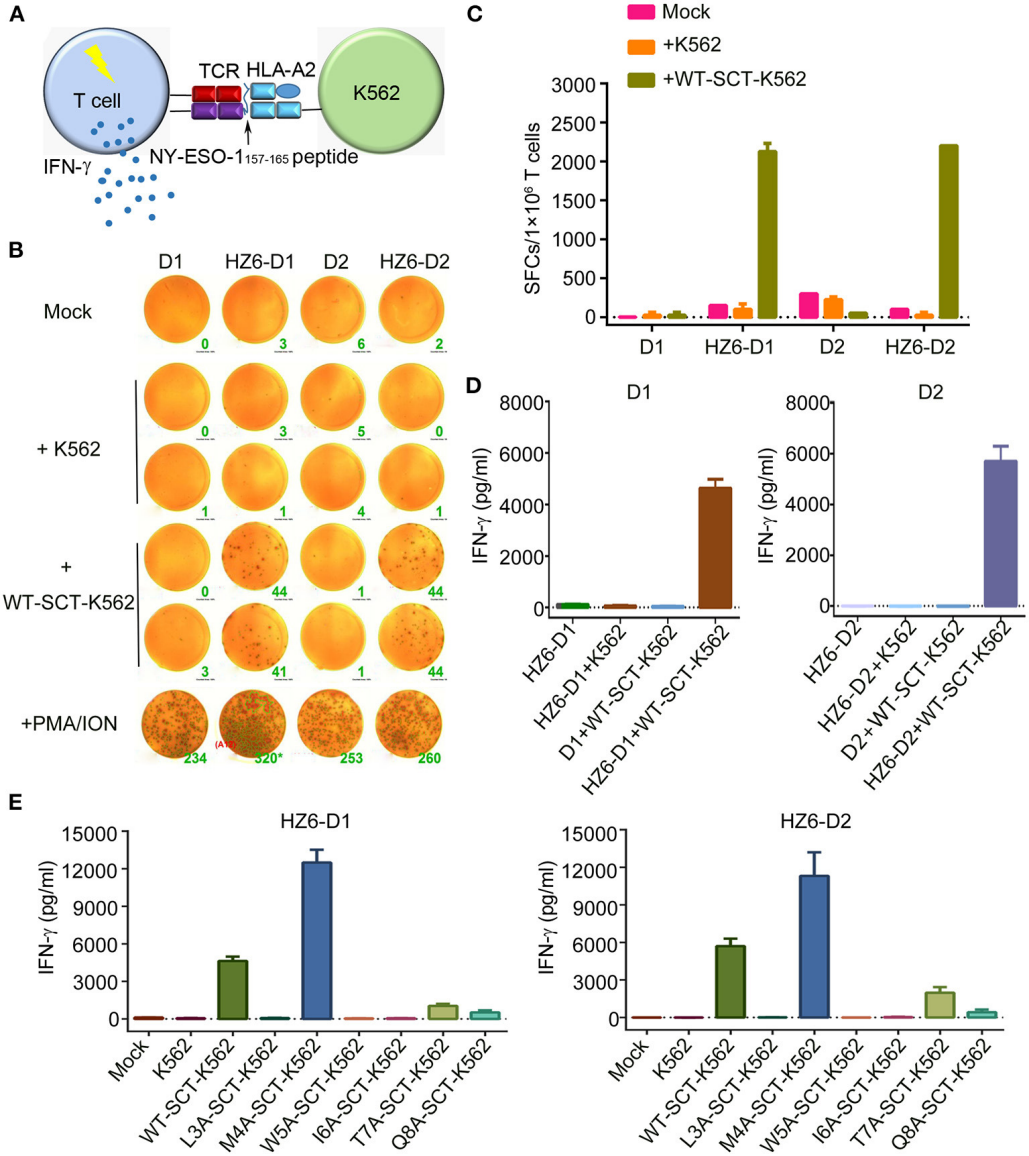
Function evaluation of HZ6 TCR-engineered primary T cells. **(A)** Schematic of the function evaluation assay in primary T cells for HLA-A2-restricted and NY-ESO-1_157−165_ specific TCRs. Primary T cells transduced TCRs were used as effector cells, and K562 cells transduced NY-ESO-1_157−165_ SCT were utilized as target cells. The reactivity of HZ6 TCR was evaluated by detecting IFN-γ secretion after co-culturing of effector cells and target cells. **(B,C)** Detection of IFN-γ-producing HZ6 TCR transduced primary T cells upon stimulating with SCT-K562 cells using ELISPOT assay. Primary T cells of 2 donors, D1 and D2, were enrolled in this experiment. **(B)** The spot forming cells (SFCs) were shown and the green number on the lower right of each well was SFCs number per 2 × 10^4^ T cells. **(C)** Statistical analysis of IFN-γ secreting HZ6 TCR transduced primary T cells stimulated with NY-ESO-1_157−165_ SCT-K562 cells or with medium alone as mock based on the results in **(B)**. **(D)** ELISA measuring secretion of IFN-γ from HZ6 TCR transduced or non-transduced primary T cells following 48 h co-incubation with WT-SCT-K562 or control K562 cells. Means ± SD for 2 technical replicates are shown. **(E)** Alanine scanning approach for HZ6 TCR in primary T cells from 2 donors (D1 and D2). HZ6-D1 T cells or HZ6-D2 T cells were co-cultured with WT-SCT-K562 or alanine-substituted pMHC-SCTs-K562 (SCT-K562 mutants) cells. IFN-γ concentrations in the supernatant were measured by ELISA. Means ± SD for 2 technical replicates are shown.

To evaluate the function of TCRs in the primary T cells, which may be applied in clinical applications, primary T cells from peripheral blood lymphocytes of two healthy donors, D1 and D2, were isolated and transduced with HZ6-TCR expressing lentivirus to generate HZ6 TCR-T cells. The lentivirus titer of the HZ8 TCR construct was too low and, therefore, HZ8 was not included in the primary TCR-T cell studies. HZ6 TCR-T cells were then co-cultured with NY-ESO-1_157−165_/HLA-A2 SCT-K562 target cells and IFN-γ production was detected (Figure 4A). The results revealed that a substantial IFN-γ secretion was induced upon specific stimulation with NY-ESO-1_157−165_/HLA-A2 SCT-K562 target cells. Both the number of IFN-γ-secreting cells detected using ELISPOT assay (Figures 4B,C) and IFN-γ secretion levels tested using ELISA (Figure 4D) were similar in D1 and D2 donors. Flow cytometry analysis revealed that CD8^+^ T cells played a major role in IFN-γ production compared with CD4^+^ T cells (Supplementary Figure 3). These results indicate that primary T cells transduced with HZ6 TCR are functionally competent in inducing of IFN-γ upon recognition of target cells.

To further investigate the functional characteristics of the HZ6 TCR, HZ6 TCR transduced T cells were co-cultured with NY-ESO-1_157−165_/HLA-A2 SCT-K562 target cells with different effector/target cell ratios, i.e., 10:1, 5:1, 1:1, 1:5, and 1:10. HZ6 TCR-T cells exhibited elevations in both the proportion of IFN-γ-releasing cells and levels of IFN-γ production, along with an increased effector/target ratio in both ELISPOT assay (Supplementary Figures 4A,B) and ELISA (Supplementary Figure 4C). Therefore, HZ6 transduced primary TCR-T cells could specifically secrete IFN-γ on encountering the NY-ESO-1_157−165_/HLA-A2 SCT-K562 target cells in a dose-dependent manner.

Next, the reactivity of HZ6 TCR-T cells to pMHC-SCT mutants were investigated. After co-culturing with the SCT-K562 mutants or WT-SCT-K562 target cells, IFN-γ production levels were measured using ELISA. The results revealed that the reactivity of HZ6 TCR-T cells from the two donors against the SCT-K562 mutants was similar to one another (Figure 4E). Consistent with the reactivity profiles of HZ6 TCR-CD8 Jurkat cells, the M4A mutation did not reduce the reactivity of HZ6 TCR-T cells, while the L3A, W5A, and I6A mutations resulted in a complete loss of response. Unexpectedly, the M4A mutation resulted in a 2-fold increase in IFN-γ production compared to WT-SCT-K562 in both D1 and D2. Although the T7A and Q8A mutations exerted no substantial influence on HZ6-CD8 Jurkat reactivity, the reactivity for HZ6 engineered primary T cells in both D1 and D2 was substantially decreased compared with that against WT-SCT-K562 target cells.

### Binding Characterization of HZ6 TCR

**Figure 5:**
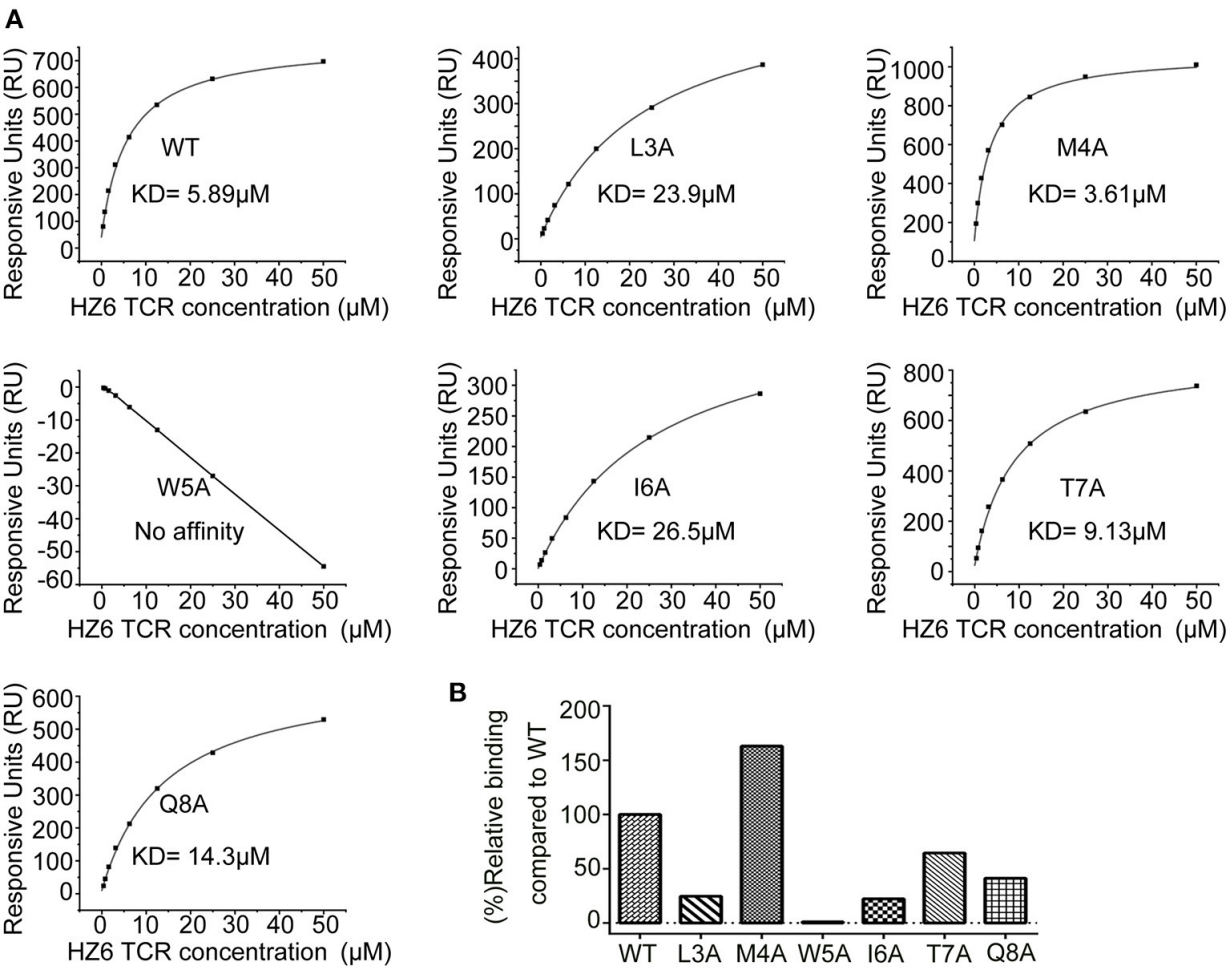
SPR characterizaiton of the binding between HZ6 TCR and WT or alanine substituted NY-ESO-1_157−165_/HLA-A2 pMHCs. **(A)** The pMHCs were immobilized on the chip and serial dilutions of HZ6 TCR were then flowed through. The figures represent measurements at equilibrium with serial 2-fold dilutions of HZ6 TCR with concentrations ranging from 50 to 0.39 μM. **(B)** The graph shows binding affinity as a percentage relative to the binding affinity to the WT peptide.

To further characterize the interaction of HZ6 TCR with NY-ESO-1_157−165_/HLA-A2, soluble HZ6 TCR proteins and pMHC proteins of HLA-A^*^0201 with WT or mutated NY-ESO-1_157−165_ peptides were prepared for surface plasmon resonance (SPR) binding assays (Supplementary Figure 5). Stable HZ6 TCR/pMHC complex proteins could be obtained when analyzed in size-exclusion chromatography after incubation of HZ6 TCR and WT NY-ESO-1_157−165_/HLA-A2 proteins, indicating a strong interaction between them (Supplementary Figure 5A). SPR analysis revealed that the binding affinity of HZ6 TCR to NY-ESO-1_157−165_/HLA-A2 (KD = 5.89 μM) is comparable to the widely used 1G4 (KD = 7.43 μM) (Figure 5A, Supplementary Figures 6, 7). T7A and Q8A mutations of NY-ESO-1_157−165_ pMHC induced a mild decrease in binding affinity to HZ6, with a KD of 9.13 and 14.3 μM, respectively. L3A and I6A mutations resulted in a substantial (4- to 5-fold) decrease in binding affinity to HZ6, while no binding to HZ6 was observed with W5A mutated NY-ESO-1_157−165_ pMHC. On the other hand, binding with the M4A mutant (KD = 3.61 μM) tended to be higher than that with WT NY-ESO-1_157−165_ pMHC. The binding affinities to HZ6 TCR ranged from high to low as follows: M4A > WT > T7A > Q8A > I6A > L3A > W5A (Figures 5A,B). This is consistent with the results of functional alanine scanning experiments with HZ6 engineered primary T cells (Supplementary Table 3).

The binding profiles of 1G4 TCR with the NY-ESO-1_157−165_ pMHC mutants was tested in parallel using the SPR assay to compare the binding patterns of HZ6 and 1G4 TCRs (Supplementary Figure 7). The results revealed that binding to 1G4 was substantially decreased with the M4A, W5A, I6A, T7A, and Q8A mutants, while the L3A mutation maintained comparable affinity to WT NY-ESO-1_157−165_ pMHC. These results suggest that the recognition pattern of HZ6 TCR to NY-ESO-1_157−165_/HLA-A2 is distinct from that of 1G4 TCR.

## Discussion

In the present study, we developed an optimized method for efficient cloning of TCRs from single T cells and identified two NY-ESO-1_157−165_ specific mTCRs from HLA-A2 transgenic mice. Both HZ6 and HZ8 were the immunodominant TCRs in their host and could specifically bind to the NY-ESO-1_157−165_/HLA-A2 complex. Functional verification with TCR-engineered Jurkat cells indicated that the recognition patterns between HZ6 and HZ8 were distinct from one another. Of note, the L3A mutation resulted in virtually no decrease in IL-2 secretion with the HZ8 TCR, while the reactivity with HZ6 was completely attenuated. HZ6 TCR-T cells were engineered using primary human T cells from two donors and were found to be competent in IFN-γ secretion on recognition with NY-ESO-1_157−165_/HLA-A2. Therefore, these TCRs may serve as promising candidates for the future development of tumor immunotherapy, though cytotoxic activity against primary tumor cells and *in vivo* tumor suppression efficacies should be further investigated.

Rapid and efficient cloning of TCRs α/β pairs is challenging for the identification of TCRs specific to TAAs. The conventional method of TCRα/β pair cloning requires the establishment of T cell clones by T cell culturing *in vitro*, which is labor-intensive, non-specific, and time-consuming (34, 40). Single-cell sequencing of TCRs can be categorized into two main approaches: the 5'-RACE method and multiplex PCR. Earlier iterations of the 5'-RACE method had a limitation in that the efficiency of PCR amplification was low (41). Although a series of improved multiplex PCR methods has been applied in cloning TCRs, multiplex PCR may generate potential PCR bias (28, 42–44). Later, the Muraguchi group improved the 5'-RACE method by adding Dynabeads Oligo (dT)_25_, resulting in a higher efficiency of PCR amplification (13–72%) (45). Here, we optimized the method to add an adaptor to the Oligo-dT primer during RT-PCR, and added an additional PCR cycle (i.e., LD-PCR) after RT-PCR to obtain sufficient and more stable, double-stranded, full-length cDNAs. The efficiency of amplifying TCRα/β pairs in Mus-177 was >80%. Thus, this optimized single-cell sequencing method was highly efficient and simple to operate for a small number of T cells, although further verification in larger samples is still needed.

Although NY-ESO-1_157−165_ is a promising target for TCR-T therapy, the number of reported specific TCRs remains limited, which may be due to difficulties in isolating TCRs from human samples. Moreover, information regarding the TCR repertoire against NY-ESO-1_157−165_ is also limited, except for the widely applied 1G4 TCR. 1G4 TCR-T cells have been evaluated in multiple clinical trials and exhibited remarkable clinical benefit in the treatment of advanced multiple myeloma and synovial cell sarcoma (19–23). The structure of the 1G4 and NY-ESO-1_157−165_/HLA-A2 complex has been reported to exhibit the recognition mechanisms of NY-ESO-1_157−165_ by 1G4 TCR (17, 46, 47). The structure of NY-ESO-1_157−165_/HLA-A2 revealed that the peptide exhibits a prominent M4-W5 “peg” in its central portion, which protrudes from the surface of the pMHC. Therefore, the protruding M4 and W5 contribute to major interactions with the 1G4 TCR and the central part of the NY-ESO-1_157−165_ peptide may determine the immunogenicity of this epitope.

The recognition patterns between HZ6 and 1G4 in binding with NY-ESO-1_157−165_, as demonstrated by SPR analysis with alanine scanning of the central residues from position 3 to 8 of NY-ESO-1_157−165_, were distinct from one another. Consistent with the structural evidence, mutation of the exposed M4 and W5 resulted in dramatically decreased binding affinity with 1G4. Although the W5A mutation also attenuated binding between HZ6 and pMHC, the M4A mutation enhanced binding between HZ6 and pMHC. The distinct recognition pattern of HZ6 to the NY-ESO-1_157−165_ mutated pMHCs was also supported by functional verification in that the M4A mutation had no substantial effects on cytokine production in HZ6 engineered Jurkat T cells or primary T cells. The L3A and I6A mutations resulted in a 5-fold decrease in binding affinity with HZ6, while HZ6 TCR-T cell reactivity to these two mutations was completely attenuated. A 2–3-fold decrease in binding affinities with T7A and Q8A resulted in substantially reduced, but still detectable, IFN-γ secretion in HZ6. There was a discrepancy between the T7A and Q8A mutants in that the responsive decrease in HZ6 engineered primary T cells was substantially larger than that in the Jurkat T cells, which may be resulting from different expression levels of TCRs on different types of T cells, or the sensitivity of cytokine production with these two cell types. Together with the binding affinities of HZ6 TCR to NY-ESO-1_157−165_ mutated pMHCs, these findings support that the functional reactivity of HZ6 is sensitive to the binding affinity for NY-ESO-1_157−165_/HLA-A2.

In summary, we identified two mTCRs specific to the NY-ESO-1_157−165_ epitope from HLA-A2 transgenic mice using an optimized single-cell sequencing method. Functional evaluations of TCR-engineered Jurkat T cells revealed distinct recognition profiles against NY-ESO-1_157−165_ epitope between HZ6 and HZ8. Further evaluation of HZ6 TCR-engineered primary T cells revealed that HZ6 TCR-T cells were competent in cytokine production upon recognition with target cells. SPR and functional analysis revealed that the functional sensitivity of HZ6 may be correlated with binding affinity between TCR and pMHC. Therefore, the mTCRs identified in the present study can broaden our understanding of the immunogenicity of NY-ESO-1_157−165_ and may serve as candidates for future development of TCR-T cell therapy.

## Materials and Methods

### Cell Lines, Murine Splenocytes

HEK-293T cells were maintained in DMEM (Gibco, Carlsbad, California) containing 10% fetal bovine serum FBS (Gibco, Carlsbad, California), 100 μg/ml streptomycin (Invitrogen, Carlsbad, California), and 100 U/ml penicillin (Invitrogen, Carlsbad, California). K562 cells and Jurkat E6-1 cells were cultured in RPMI 1640 medium (Gibco, Carlsbad, California) supplemented with 10% FBS, 100 μg/ml streptomycin (Invitrogen), and 100 U/ml penicillin (Invitrogen). Murine splenocytes were maintained in RPMI 1640 medium supplemented with 10% FBS, 100 μg/ml streptomycin, 100 U/ml penicillin, and 50 IU/ml rhIL-2 (Invitrogen, Carlsbad, California). All cells were incubated at 37°C under 5% CO_2_.

### Synthetic Peptides

All of the peptides we used in this study are summarized in Supplementary Table 1. These peptides were synthesized from Scilight Biotechnology LLC (Beijing, China). The purity of the synthesized peptides was >95%, as determined by high-performance liquid chromatography analysis. Peptides were dissolved in DMSO and diluted in PBS or RPMI 1640 medium.

### Tetramer Preparation

Human leukocyte antigen-A^*^0201-restricted tetramers with peptide NY-ESO-1_157−165_: SLLMWITQC or HPV-E6_29−38_: TIHDIILECV were prepared as previously described (48). Briefly, the extracellular domain of HLA-A^*^0201 (1-276) was modified by the addition of a substrate sequence for the biotinylation enzyme BirA at the C-terminus of the α3 domain. The modified HLA-A2 and β2-microglobulin were expressed in *Escherichia coli* and refolded in the presence of peptides. *In vitro*-renatured peptide/HLA-A2 complexes were then purified and biotinylated by incubation with D-biotin, ATP, and the biotin protein ligase BirA (Avidity, Aurora, Colorado) at 4°C for 12 h. The samples were further purified using a Superdex 200 10/300 GL gel filtration column (GE Healthcare, Chicago, Illinois). Finally, the tetramers were formed by mixing with PE-streptavidin (Sigma-Aldrich, Saint Louis, Missouri) and stored at 4°C in PBS containing 0.5 mM EDTA, 0.2% BSA, 10 mM Tris-HCI (pH 8.0), 150 mM NaCI, and 0.09% NaN_3_.

### Flow Cytometry

The following conjugated antibodies were used in this study: anti-mCD3 (17A2, BioLegend, San Diego, California, cat. no. 100204), anti-mCD8 (53-6.7, BioLegend, San Diego, California, cat. no. 100734), anti-hTCRα/β (IP26, BioLegend, San Diego, California, cat. no. 306723), anti-hCD3 (HIT3α, BioLegend, San Diego, California, cat. no.300325), anti-hCD8 (HIT8α, BioLegend, San Diego, California, cat.no.300905), anti-hCD4 (OKT4, BioLegend, San Diego, California, cat. no. 317431), anti-HLA-A2 (BB7.2, BioLegend, San Diego, California, cat. no. 343303), and anti-hIFNγ (B27, BioLegend, San Diego, California, cat. no. 506517). Staining with the fluorescent antibodies and pMHC tetramers were performed in FACS buffer (2% FBS in PBS) at room temperature. Cells were stained with 0.05 μg/μl tetramer per 1 × 10^6^ cells for 30 min, followed by other fluorescent antibodies. Antibodies diluted at a 1:100 ratio in FACS buffer were stained for 20 min. Samples were analyzed on FACS Calibur (BD Biosciences, San Jose, California) and FACS Aria (BD Biosciences, San Jose, California). Data analysis was performed using FlowJo software (Tree Star, Ashland, Oregon).

### Immunization of HLA-A^*^0201 Transgenic Mice

Transgenic mice expressing the human HLA-A^*^0201 gene were obtained from Jackson Lab and housed in specific pathogen free (SPF) mouse facilities. All animal experiments were approved by the Committee on the Ethics of Animal Experiments of the Institute of Microbiology, Chinese Academy of Science (IMCAS), conducted as previously described, and in compliance with the recommendations in the Guide for the Care and Use of Laboratory Animals of IMCAS Ethics Committee. Briefly, 8- to 12-week old mice were immunized subcutaneously with 100 μg of HLA-A^*^0201-restricted peptide NY-ESO-1_157−165_ (SLLMWITQC) or an equal volume of PBS as control, plus 120 μg of HBVc 128-140 helper peptide, emulsified in 100 μl of incomplete Freund's adjuvant (Sigma, Saint Louis, Missouri), as described before (34, 49). A booster immunization was given using complete Freund's adjuvant (Sigma, Saint Louis, Missouri) instead of incomplete Freund's adjuvant 1 week later. One week after the booster immunization, mice were euthanized, and splenocytes were harvested and cultured.

### Single-Cell Sequencing of TCRs and TCR Expressing Constructs

#### RT-PCR

One day after murine splenocytes culture, splenocytes were labeled with anti-mCD3, anti-mCD8, and NY-ESO-1_157−165_/HLA-A2 tetramer. Tetramer^+^/CD8^+^ T cells were single-cell-sorted into each well of 96-well PCR plate on a FACS Aria sorter and then reverse transcribed as reported before (38). In brief, a single cell with cell lysis buffer, Oligo(dT) with adaptor sequence, and dNTP mix were incubated at 72°C. Then, the mixtures were added into another solution containing M-MLVRTase, RT buffer, RNAse inhibitor, Betaine, DTT, and MgCl_2_, TSO primer in a 20 μL reaction.

#### Long-Distance-PCR

The following solutions of adaptor primer, dNTP mix, Phusion HF Buffer, and Phusion DNA Polymerase (Thermo Scientific, Waltham, Massachusetts) were added into the 20-μl reactions above to obtain sufficient and more stable double-strand full-length cDNAs.

#### Nested PCR

Nested PCR was performed with adaptor primers and TCR constant region primers as reported before (50).

PCR products were then analyzed on a 1.0% agarose gel, purified, and sequenced. TCR sequences were analyzed with the IMGT/V-Quest tool (http://www.imgt.org/).

After sequencing and confirming, we amplified the leader and variable region of TCR α or β chain using the template above. Then, we got chimeric products of “murine variable region+ human constant region” using overlapping PCR. The chimeric TCR chains were subcloned into a pCDH-EF1-MCS-T2A-Puro vector in a final format of “chimeric TCRα-P2A-chimeric TCRβ-T2A-Puro” through several PCRs and restriction enzyme reactions.

### CD3-CD8 and pMHC-SCT Constructs

We designed the CD3-CD8 construct in the format of hCD3δ-F2A-hCD3γ-T2A-hCD3ε-E2A-hCD3ζ-IRES-hCD8α as previously reported (51). pMHC SCTs of WT-SCT: NY-ESO-1_157−165_/A2, L3A-SCT: NY-ESO-1_157−165_-L3A/A2, M4A-SCT: NY-ESO-1_157−165_-M4A/A2, W5A-SCT: NY-ESO-1_157−165_-W5A/A2, I6A-SCT: NY-ESO-1_157−165_-I6A/A2, T7A-SCT: NY-ESO-1_157−165_-T7A/A2, and Q8A-SCT: NY-ESO-1_157−165_-Q8A/A2 were prepared with a disulfide trap modification as described before (52). CD3-CD8 and pMHC SCTs genes were synthesized from Synbio Technologies (Suzhou, China) and cloned into a pCDH-EF1-MCS-T2A-Puro vector.

### Evaluation of TCR Surface Expression and Specificity in Transfected 293T Cells

HEK-293T cells were plated on six-well-plates a day prior to transfection. Cells in each well were transfected transiently with 1.25 μg TCR construct plasmid and 1.25 μg CD3-CD8 construct plasmid. The medium was refreshed after 6 h. Cells were stained with anti-TCRα/β, anti-hCD3, and NY-ESO-1_157−165_/HLA-A2 tetramer-PE or HPV-E6/HLA-A2-tetramer-PE and analyzed by flow cytometry 48 h after transfection.

### Lentiviruses Production and Concentration

Lentiviruses carrying TCR, CD3-CD8, and pMHC SCTs were produced separately with HEK-293T cells. In brief, 1 day prior to transfection, 293T cells were plated on 15 cm plates. Cells in each plate were transfected with 20 μg pLP1, 13 μg pLP2, 5 μg VSVG, and 20 μg TCR construct expressing plasmid using PEI transfection reagent. DMEM containing 2% FBS, 100 μg/ml streptomycin, 100 U/ml penicillin, and 0.12% Sodium butyrate was replaced after 12 h. High-titer lentiviruses were obtained by collecting the supernatants above after 48 h of transfection and concentrated 50-fold by using the PEG-8000 precipitation method.

### Functional Assays in Jurkat T Cells or Primary T Cells

Jurkat T cells or K562 cells were plated on a six-well-plate a day prior to transduction. TCR-CD8-Jurkat cells were prepared by adding high-titer TCR and CD3-CD8 lentiviruses into the Jurkat cells, together with 12 μg/ml protamine. SCT-K562 cells were obtained by adding high-titer pMHC-SCT lentiviruses into K562 cells along with 12 μg/ml protamine. To improve the expression of TCR and CD8, TCR-CD8-Jurkat cells were cultured for 14 days under the pressure of 2 μg/ml puromycin. TCR-CD8-Jurkat cells were co-incubated in a 96-well-plate at a 1:1 ratio with various SCT-K562 cells. After 48 h of co-incubation, the supernatant was tested for secreted IL-2 with an ELISA kit and processed according to the protocol of the manufacturer (Biolegend, San Diego, California).

Human peripheral blood lymphocytes (PBLs) from healthy donors (D1 and D2) were used. PBLs were isolated from heparinized blood by density gradient centrifugation using Ficoll-Hypaque (Tianjin Haoyang). T cells were isolated from PBLs with magnetic beads coated with anti-CD4 and anti-CD8 antibodies and processed according to the protocol of the manufacturer (MACS). Two days before viral transduction, six-well-plates were coated with RetroNectin (Takara Bio, Japan) for 2 h. 1–2 × 10^6^ total primary T cells were added to the pre-coated plates and activated per well using the Human T-Activator CD3/CD28 (ThermoFisher, Waltham, MA) in GT-T551 T cell medium (Takara, Japan) supplemented with 5% (v/v) human AB serum, 2,000 U/ml rhIL-2 and antibiotics (pen/strep). After activation, high-titer TCR-lentiviruses and protamine sulfate (12 μg/ml) were added into the activated primary T cells. The medium was refreshed 24 h later and maintained with GT-T551 T cell medium. After 7 days, transduced T cells were assayed for TCR surface expression and used for functional assays as below.

#### Intracellular Cytokine Staining

Intracellular cytokine staining assay was performed as previously described (53). Briefly, TCR-transduced T cells were co-cultured with SCT-K562 cells at a ratio of 1:1 in 96-well-plates for 4 h. Cells cultured with medium alone or PMA/ION were used as negative or positive controls, respectively. The cells were incubated with Golgistop (BD Biosciences, San Jose, California) for an additional 10 h at 37°C. Cells were stained with anti-CD3 and anti-CD4/or anti-CD8 surface markers, fixed, and permeabilized in permeabilizing buffer (BD Biosciences, San Jose, California), and subsequently stained with anti-IFN-γ-PE/Cy7. Samples were then washed, re-suspended, and then analyzed on a FACSAria III.

#### Interferon γ-Specific ELISPOT Assay

ELISPOT assay was performed using an IFN-γ secreting ELISPOT assay (BD Bioscience, San Jose, California) as previously described (53). In brief, 96-well ELISPOT plate was pre-incubated with anti-IFN-γ coating antibody overnight at 4°C, and then blocked for 2 h at RT. With TCR-transduced T cells as effector cells and SCT-K562 cells as target cells, the effector cells and target cells were co-incubated in pre-incubated plates with different ratios (10:1; 5:1; 1:1; 1:5; and 1:10) with a total of 2 × 10^4^ cells per well. Effector cells incubated with PMA/ION were added as positive controls, and effector cells incubated with medium were employed as negative controls. After incubation of 18 h, cells were removed, and the plates were processed according to the instructions of the manufacturer (BD Life Sciences, San Jose, California). The number of spots were determined using an automatic ELISPOT reader and image analysis software (Cellular Technology Ltd., Ohio).

#### Interferon γ-Specific ELISA Assay

The effector cells and SCT-K562 target cells were co-incubated in the 96-well-plates at different ratios (10:1; 5:1; 1:1; 1:5; and 1:10), while the effector cells and SCT-K562 target cells carrying alanine substituting NY-ESO-1_157−165_ mutants were co-incubated at a 1:1 ratio. After 48 h of co-incubation, the supernatant was tested with a commercial ELISA kit for secreted IFN-γ (BioLegend, San Diego, California).

### Surface Plasmon Resonance Assay

For SPR analysis, TCR protein expression and purification were conducted as reported before (54, 55). Briefly, the variable region of murine TCR and the ectodomains of human TCR constant genes with an artificial disulfide bond were cloned into pET21a (Invitrogen, Carlsbad, California). TCR α and β chain proteins were expressed separately as inclusion bodies in *E.coli* (BL21-DE3). Soluble TCR proteins were obtained by an *in vitro* refolding process. The dissolved α- and β-chain inclusion bodies were injected into refolding buffer containing 5 M urea, 400 mM L-arginine HCl, 100 mM Tris (pH 8.0), 5 mM reduced glutathione, and 0.5 mM oxidized glutathione. The refolding mixture was then dialyzed for 24 h in 10 volumes of ultrapure water (Milli-Q system) and then against 10 volumes of 10 mM Tris/10 mM NaCl (pH 8.0). The refolded samples were loaded on a Source 15Q anion exchange column (GE Healthcare, Chicago, Illinois) and further purified by gel filtration on a Superdex 200 10/30 GL column (GE Healthcare, Chicago, Illinois). Purified proteins were analyzed by reduced (with Dithiothreitol) and non-reduced (without Dithiothreitol) SDS-PAGE analysis.

The expression and purification procedure of biotinylated pMHCs with seven various peptides were similar to that of Tetramer preparation above, but without adding PE-streptavidin to form tetramer. TCR and biotinylated pMHCs proteins were finally buffer exchanged into HBS-P buffer containing 10 mM HEPES (pH 7.4), 150 mM NaCl, and 0.005% [vol/vol] surfactant P20. Binding analysis was performed on a Biacore 8K machine with streptavidin chips (SA chips; Biacore). Approximately 300 to 500 response units of biotinylated pMHC proteins were immobilized on the chip. A series of 2-fold diluted TCR proteins ranging from 0.39 to 50 μM was then flowed over the chip surface. The Multi-cycle binding kinetics was analyzed using the Biacore 8K Evaluation Software (version1.1.1.7442) using a1:1 Langmuir binding model.

## Data Availability Statement

The original contributions presented in the study are included in the article/Supplementary Material, further inquiries can be directed to the corresponding author/s.

## Ethics Statement

The animal study was reviewed and approved by Committee on the Ethics of Animal Experiments of the Institute of Microbiology, Chinese Academy of Science (IMCAS).

## Author Contributions

ST and GG planned the study. HZ, ST, and GG have made substantial contributions to the conception and design of the study, execution of the experiments, and preparation of the manuscript. HZ, ST, GG, MS, JW, BZ, XC, and YH have involved in the analysis and interpretation of data. All authors provided the final approval of the version to be published, agreed to be accountable for all aspects of the research by ensuring that the questions relating to the accuracy or integrity of any part of the study are appropriately investigated and resolved, contributed to the article, and approved the submitted version.

## Conflict of Interest

The authors declare that the research was conducted in the absence of any commercial or financial relationships that could be construed as a potential conflict of interest.
