# Supplementary material for: Identification of NY-ESO-1157–165 Specific Murine T Cell Receptors With Distinct Recognition Pattern for Tumor Immunotherapy
Source: Front Immunol. 2021 Mar 23;12:644520. doi: 10.3389/fimmu.2021.644520 (PMC8021954; doi:10.3389/fimmu.2021.644520)
Supplement: Supplementary file 1 [file Data_Sheet_1.PDF]

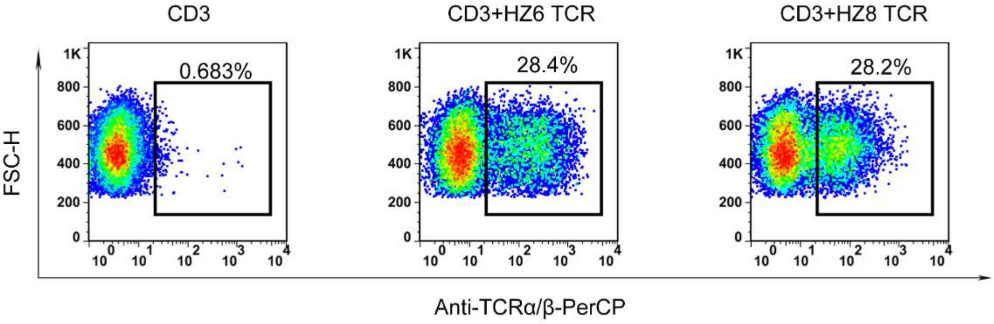

Figure S1. HZ6 and HZ8 TCRs expression in HEK-293T cells system. Cloned TCRs and human CD3-CD8 complex were co-transfected into 293T cells. TCR expression on the surface of 293T cells was detected by staining with anti-TCRα/β antibodies and analyzed with flow cytometry.

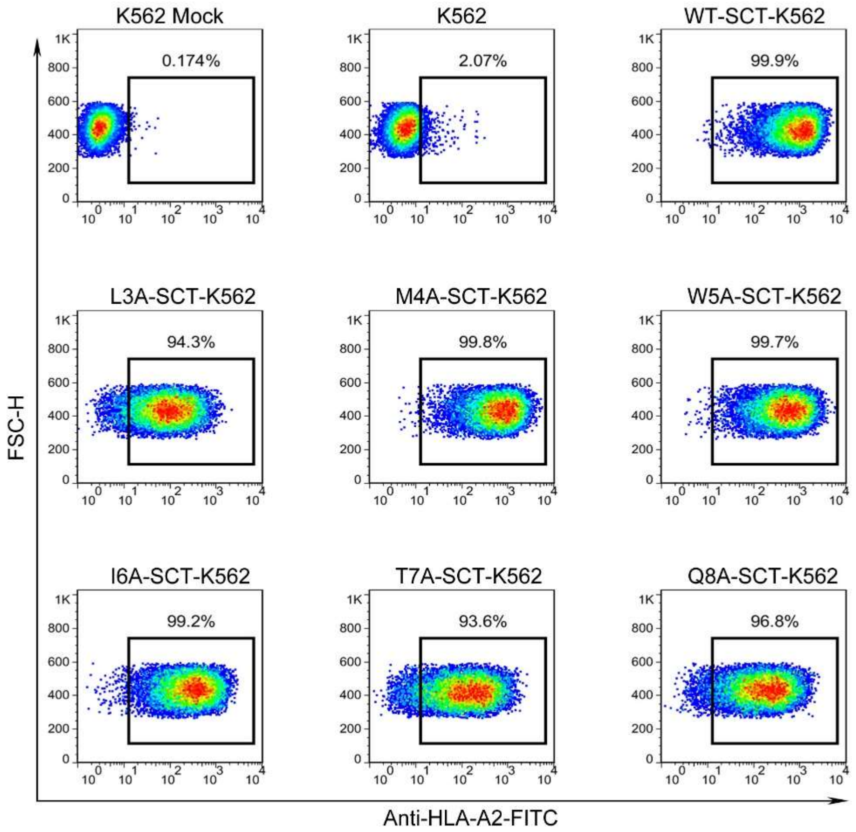

Figure S2. Flow cytometry analysis of K562 cells stably expressing WT- NY-ESO-1$_{157\text{-}165}$ or alanine-substituted pMHC-SCTs (SCT-K562 mutants). K562 cells were transduced with NY-ESO-1$_{157\text{-}165}$ pMHC SCT lentiviruses, either WT-SCT or SCT mutants carrying alanine-substituted NY-ESO-1$_{157\text{-}165}$. Transduction and expression efficiency of pMHC SCTs on K562 cells were detected by staining with anti-HLA-A2 antibodies.

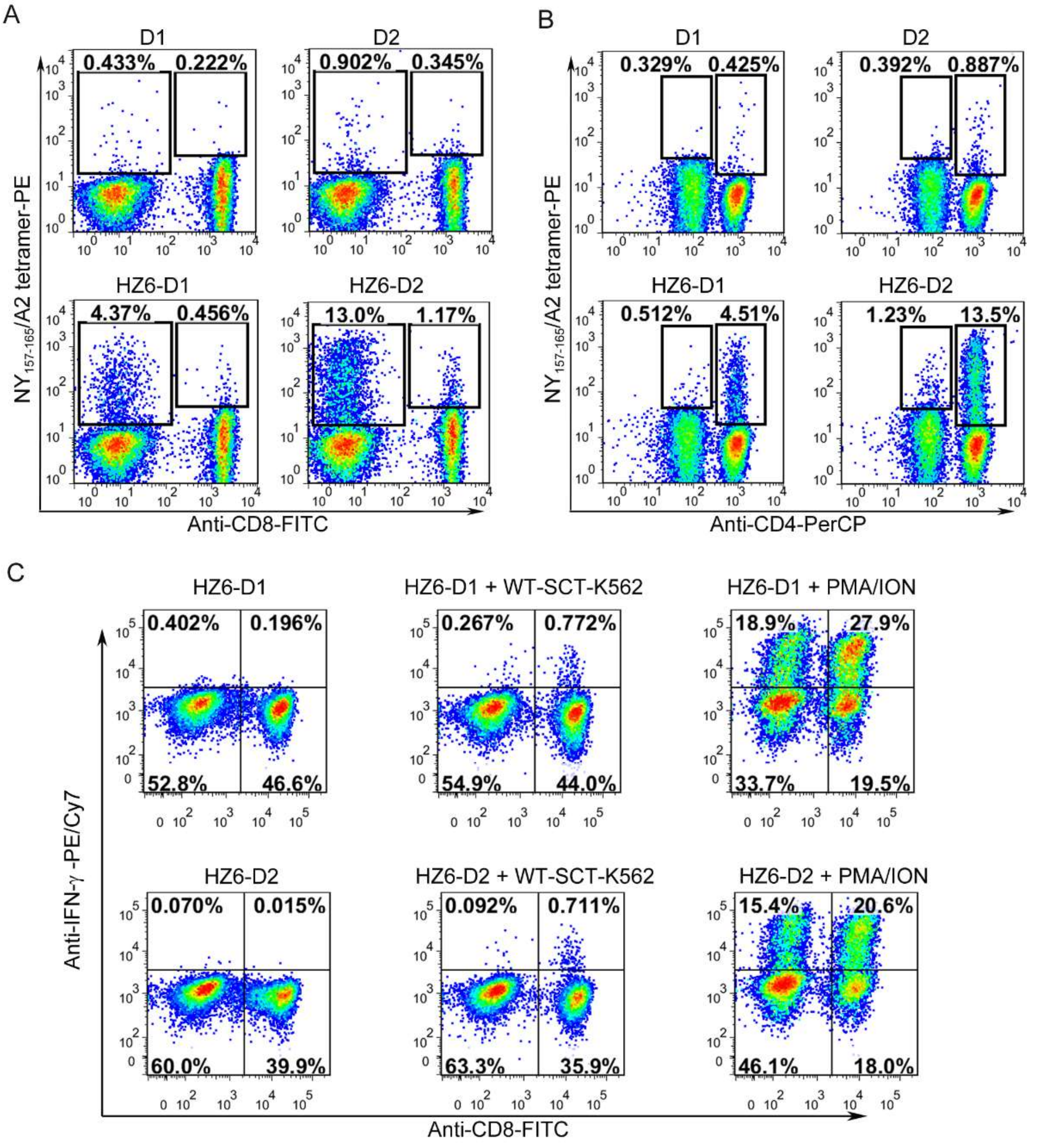

Figure S3. HZ6 TCR transduction efficiency and the percentage of IFN-γ-secreting T cells in HZ6 TCR transduced primary T cells. Primary T cells of two donors, D1 and D2, were transduced with HZ6 TCR lentivirus to generate HZ6 TCR-T cells (HZ6-D1 and HZ6-D2). (A-B) Flow cytometry analysis of HZ6 TCR transduction and expression efficiency in $CD8^+$ T cells (A) and $CD4^+$ T cells (B). (C) Flow cytometry analysis of IFN-γ-secreting T cells in HZ6 TCR transduced T cells. HZ6-D1 or HZ6-D2 T cells were co-cultured with WT-SCT-K562 cells, PMA/ION (positive control) or with medium alone as mock. IFN-γ- secreting T cells were detected

by Intracellular cytokine staining (ICS).

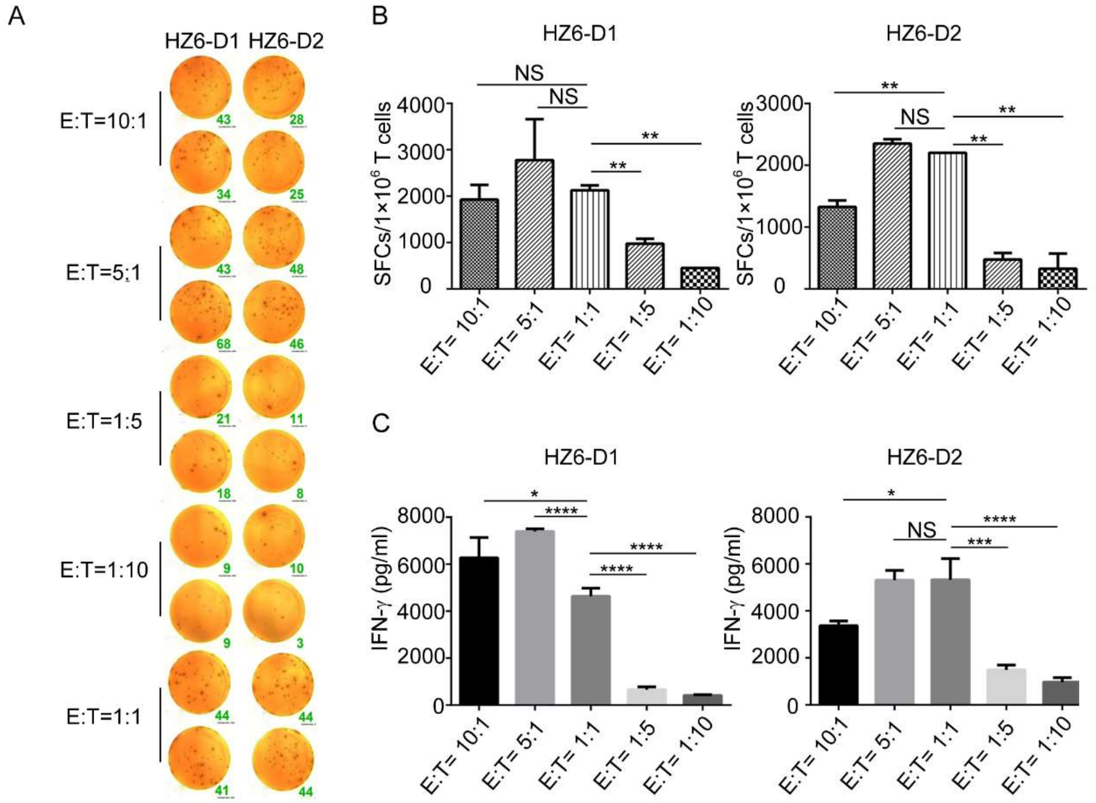

Figure S4. HZ6 transduced primary TCR-T cells secret IFN-γ upon encounter with the WT-SCT-K562 target cells in a dose-dependent manner. HZ6-D1 or HZ6-D2 T cells were co-cultured with WT-SCT-K562 cells with different effector/target (E/T) cell ratios, *i.e.*, 10:1, 5:1, 1:1, 1:5 and 1:10. (A-B) Detection of IFN-γ secretion from HZ6 TCR transduced primary T cells following 48h co-incubation with WT-SCT-K562 cells by ELISPOT assay. (A) The spot forming cells (SFCs) are shown. The green number on the lower right of each well was SFCs number per 2×10^4 T cells. (B) Statistical results of SFCs number per 1×10^6 T cells based on the results of sup Fig. 3A. (C) IFN-γ secretion measurement of HZ6 TCR transduced primary T cells by ELISA assay. Means ± SD for 4 technical replicates are shown. In (B) and (C), t test was

performed for statistical analysis. NS (not significant), $*P \leqslant 0.05$, $**P \leq 0.01$, $***P \leq 0.001$, $****P \leq 0.0001$.

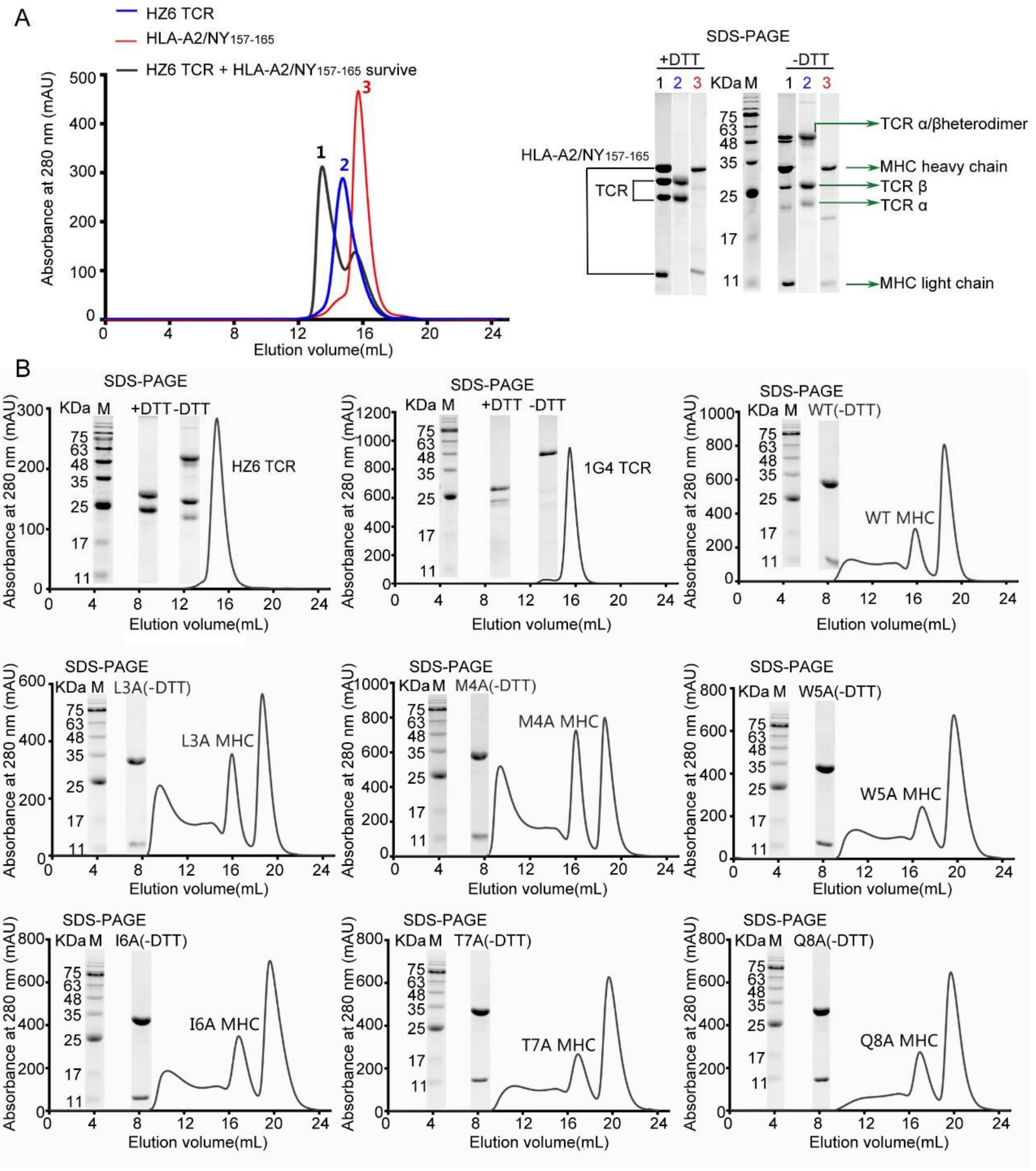

Figure S5. Purification of soluble TCRs and pMHCs proteins using a Superdex 200 10/300 GL column. The X axis represents the elution volume of each protein. M, molecular weight marker. (A) Gel filtration profiles of the HZ6 TCR, HLA-A2/NY-ESO-1$_{157\text{-}165}$ (NY$_{157\text{-}165}$) and HZ6 TCR/ NY$_{157\text{-}165}$/HLA-A2 complex were analyzed by

size-exclusion chromatography as indicated. The HZ6 TCR/HLA-A2/$NY_{157-165}$ complex can form stable complex proteins on the gel filtration column. On the right was SDS-PAGE analysis of the HZ6 TCR/HLA-A2 /$NY_{157-165}$ complex protein. The presence of HZ6 TCR α and β chain bands and HLA-A2 /$NY_{157-165}$ heavy and light chain bands in line 1 supports the formation of HZ6 TCR/HLA-A2 /$NY_{157-165}$ complex. (B) Gel filtration profiles and SDS-PAGE assay confirmation of soluble HZ6 and 1G4 TCR proteins and NY-ESO-$1_{157-165}$ pMHC proteins of HLA-A2 with WT- or alanine-substituted NY-ESO-$1_{157–165}$ peptides.

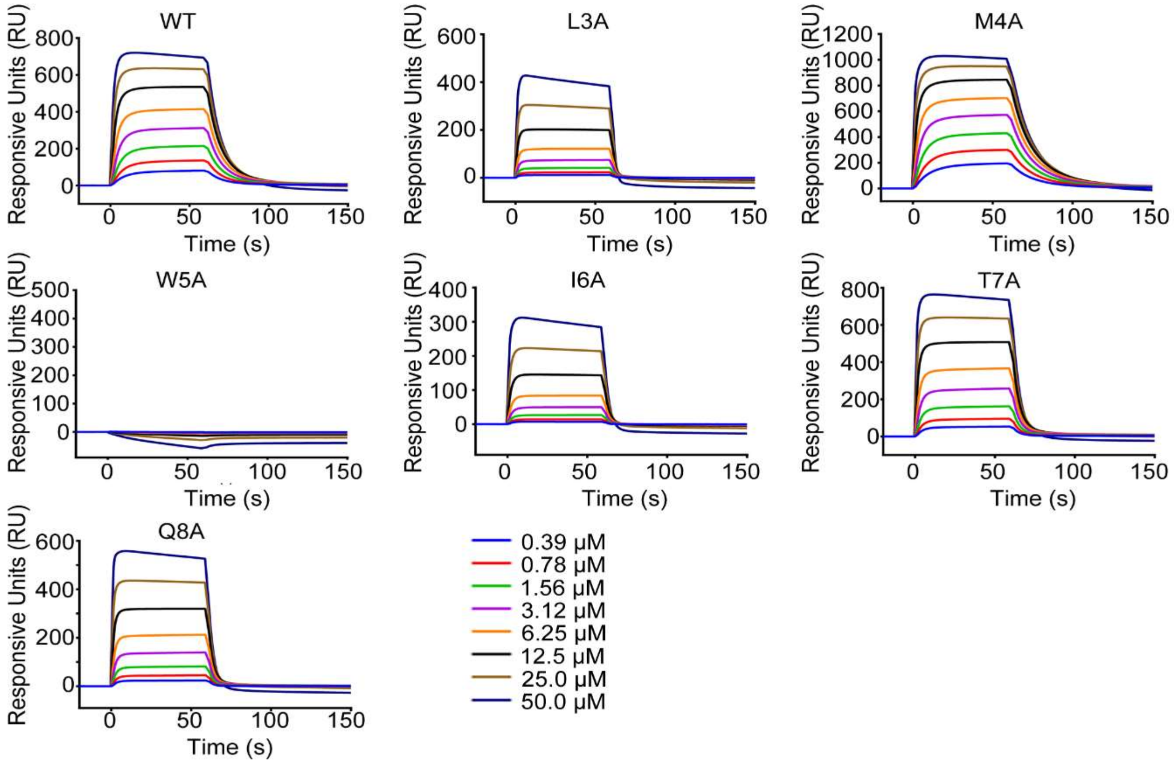

Figure S6. Binding profiles of soluble HZ6 TCR and WT- or alanine-substituted NY-ESO-1$_{157\text{-}165}$/HLA-A2 by kinetic measurements of SPR. The pMHCs of HLA-A*0201 loaded with WT- or alanine-substituted NY-ESO-1$_{157–165}$ peptides from position 3-8 (L3A, M4A, W5A, I6A, T7A, Q8A) were immobilized on the chip and serial dilutions of HZ6 TCR were then flowed through. The figures represent kinetic measurements with serial two-fold dilutions of HZ6 TCR with concentrations ranging from 50 μM to 0. 39 μM.

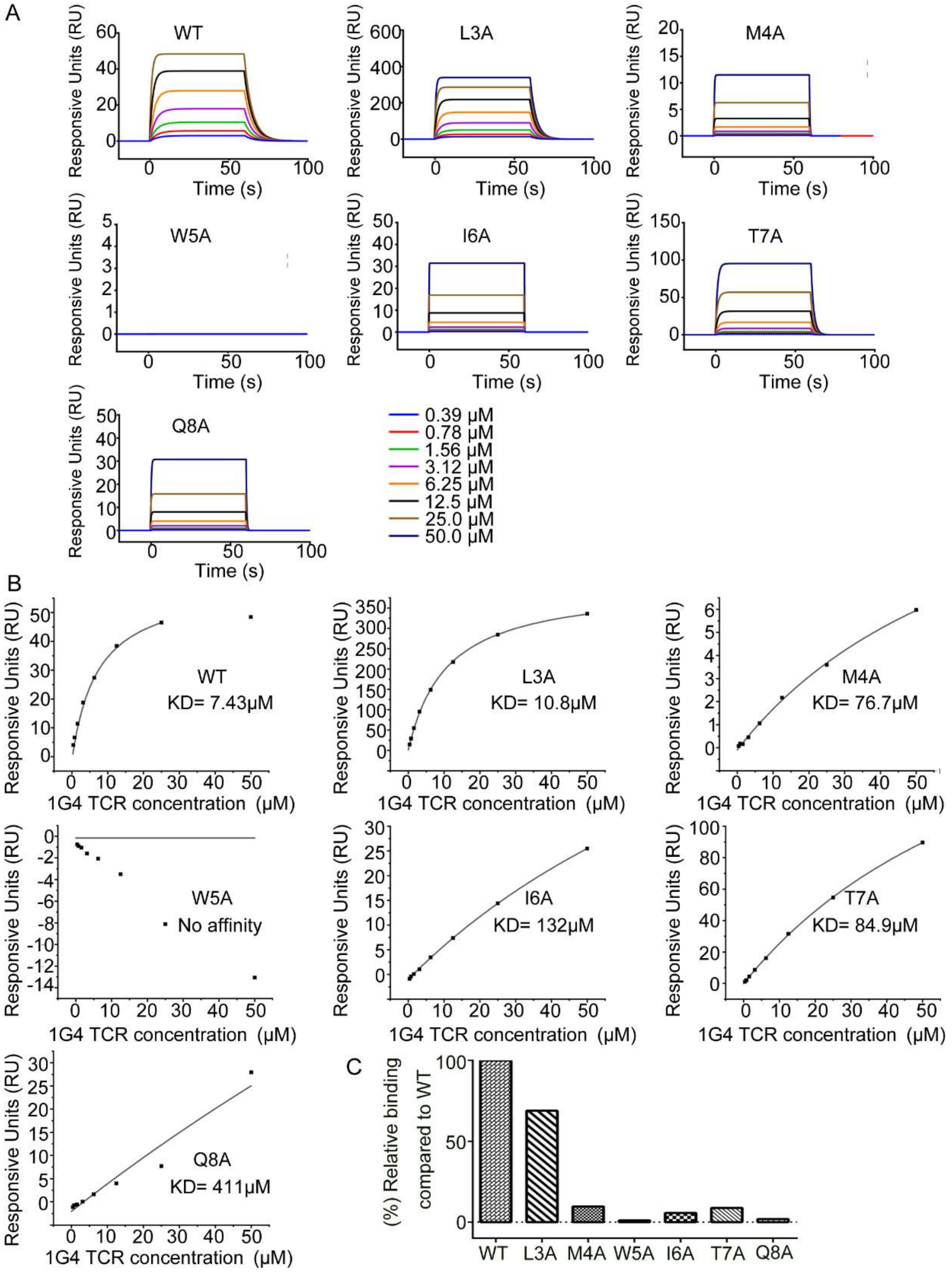

Figure S7. SPR analysis of the binding of soluble 1G4 TCR to pMHC proteins of HLA-A*0201 with WT or alanine-substituted NY-ESO-1$_{157–165}$ peptides from position 3 to 8 (L3A, M4A, W5A, I6A, T7A, Q8A). The pMHCs were immobilized on the chip and serial two-fold dilutions of 1G4 TCR ranging from 50 μM to 0. 39 μM

were then flowed through. (A) The figures show kinetic measurements. (B) The figures represent measurements at equilibrium. (C) The graph shows binding affinity as a percentage relative to the binding affinity to the WT peptide.

Table S1. Peptides used in this study

Peptide name	Amino acid sequence
NY-ESO-1 $_{157-165}$	SLLMWITQC
NY-ESO-1 $_{157-165}$-L3A	SL**A**MWITQC
NY-ESO-1 $_{157-165}$-M4A	SLL**A**WITQC
NY-ESO-1 $_{157-165}$-W5A	SLLM**A**ITQC
NY-ESO-1 $_{157-165}$-I6A	SLLMW**A**TQC
NY-ESO-1 $_{157-165}$-T7A	SLLMWI**A**QC
NY-ESO-1 $_{157-165}$-Q8A	SLLMWIT**A**C
HBVc $_{128-140}$ helper peptide:	TPPAYRPPNAPIL
HPV-E6 $_{29-38}$	TIHDIILECV

Table S2. Analysis of TCR repertoire from Mus-2 and Mus-177

Clone name	CDR3β	TRBV	TRBJ	CDR3α	TRAV	TRAJ	Clone number/ Frequency	CD3α Frequency	CD3β Frequency
2-A6, etc	ASSEGGNTLY	TRBV13-3*01	TRBJ1-3*01	IVTDHTNAYKVI	TRAV2*01	TRAJ30*01	4/40%	100%	70%
2-A7, etc	ASSEGGNTLY	TRBV13-3*01	TRBJ1-3*01	Amplification failure			3/30%	---	
2-A3	ASSLANSDYT	TRBV12-2*02	TRBJ1-2*01	Amplification failure			1/10%	---	10%
2-C11	ASSLALEVF	TRBV16*01	TRBJ1-1*01	Amplification failure			1/10%	---	10%
2-A5	ASSDAGTGDEQY	TRBV13-1*02	TRBJ2-7*01	Amplification failure			1/10%	---	10%
177-B5	ASSDWGNYAEQF	TRBV13-3*01	TRBJ2-1*01	MSYASSGSWQLI	TRAV16*01	TRAJ22*01	1/2.13%		2.22%
177-A7	ASSQEHTYEQY	TRBV2*01	TRBJ2-7*01	MSYASSGSWQLI	TRAV16*01	TRAJ22*01	1/2.13%	82.90%	2.22%
177-A1,etc	ASSIVFQDTQY	TRBV19*01	TRBJ2-5*01	MSYASSGSWQLI	TRAV16*01	TRAJ22*01	32/68.10%		
177-B7	ASSIVFQDTQY	TRBV19*01	TRBJ2-5*01	ASGSAGNKLT	TRAV14D-3	TRAJ17*01	1/2.13%	2.44%	82.20%
177-A6	ASSIVFQDTQY	TRBV19*01	TRBJ2-5*01	MREGQGTGSKLS	TRAV16D	TRAJ58*01	1/2.13%	2.44%	
177-A2, etc	ASSIVFQDTQY	TRBV19*01	TRBJ2-5*01	Amplification failure			3/6.38%	---	
177-A8	ASSQDGGANSDYT	TRBV2*01	TRBJ1-2*01	Amplification failure			1/2.13%	---	2.22%
177-B3	ASSSSRDRVNERLF	TRBV3*01	TRBJ1-4*02	Amplification failure			1/2.13%	---	2.22%
177-D4	ASSLIGTSSQNTLY	TRBV3*01	TRBJ2-4*01	ALRGTQVVGQLT	TRAV6N-6*01	TRAJ5*01	1/2.13%	2.44%	2.22%
177-A11	ASGDDWGGSAETLY	TRBV13-2*01	TRBJ2-3*01	Amplification failure			1/2.13%	---	2.22%
177-C7	TCSADRGLNYAEQF	TRBV1*01	TRBJ2-1*01	AVLTGNTGKLI	TRAV7D-3*01	TRAJ37*01	1/2.13%	2.44%	2.22%
177-B12	ASSRDSNYAEQF	TRBV4*01	TRBJ2-1*01	AGDTNAYKVI	TRAV10*02	TRAJ30*01	1/2.13%	2.44%	2.22%
177-B8	Amplification failure			MRASSGSWQLI	TRAV16N*01	TRAJ22*01	1/2.13%	2.44%	---
177-D2	Amplification failure			VLSYNTNTGKLT	TRAV9N-2*01	TRAJ27*01	1/2.13%	2.44%	---

Table S3. Fold-changes of cytokine production or binding affinity of HZ6, HZ8 or 1G4 to NY-ESO-1$_{157–165}$ mutants compared with wild type peptide

	L3A	M4A	W5A	I6A	T7A	Q8A
HZ6-CD8 Jurkat cell [1]	0.010	0.963	0.008	0.079	0.939	0.825
HZ8-CD8 Jurkat cell [1]	0.888	0.478	0.004	0.048	0.029	0.829
HZ6-primary T D1[2]	0.016	2.700	0.010	0.012	0.225	0.112
HZ6-primary T D2 [2]	0.001	1.983	0.001	0.006	0.347	0.073
Soluble HZ6 [3]	0.246	1.631	NO[4]	0.222	0.645	0.412
Soluble 1G4 [3]	0.690	0.097	NO[4]	0.056	0.088	0.018

[1] Responses of Jurkat cells transduced with HZ6 or HZ8 TCR to the K562 cells carrying alanine substituted NY-ESO-1$_{157–165}$ SCT. The data represents the ratio of IL-2 secretion level against K562 carrying SCT mutants to that against wild type SCT-K562 cells.

[2] Responses of primary T cells from D1 or D2 transduced with HZ6 or HZ8 TCR to the K562 cells carrying alanine substituted NY-ESO-1$_{157–165}$ SCT. The data represents the ratio of IFN-γ secretion level against K562 carrying SCT mutants to that against wild type SCT-K562 cells.

[3] Binding affinity (KD) of HZ6 or 1G4 with alanine substituted or wild type NY-ESO-1$_{157–165}$ pMHC. The data represents the ratio of binding affinity (KD) of the corresponding TCR with pMHC mutants to that with wild type NY-ESO-1$_{157–165}$ pMHC.

[4] NO, not observed.
